# Chemical Composition and Antimicrobial Activities of Iranian Propolis

**DOI:** 10.22034/ibj.22.1.50

**Published:** 2018-01

**Authors:** Houshang Afrouzan, Azar Tahghighi, Sedigheh Zakeri, Ali Es-haghi

**Affiliations:** 1 Malaria and Vector Research Group (MVRG), Biotechnology Research Center (BRC), Pasteur Institute of Iran, Tehran, Iran; 2 Honey Bee Department Animal Sciences Research Institute, Karaj, Iran; 3 Department of Physico Chemistry, Razi Vaccine and Serum Research Institute Karaj, Iran

**Keywords:** Propolis, Gas chromatography-mass spectrometry, Flavonoids, Iran

## Abstract

**Background:**

With considering the importance of natural products for their remedial and therapeutic value, this research was aimed to analyze the chemical compositions and antimicrobial activity of four propolis samples from different areas of Iran (Chenaran, Taleghan, Morad Beyg, and Kalaleh) with various climates and flora.

**Methods:**

Ethanolic (70% EtOH) and dichlromethane (DCM) extracts of Iranian propolis were analyzed by gas chromatography-mass spectrometry (GC-MS) methods, and antimicrobial activity was evaluated against *Candida albicans, Escherichia coli*, and *Staphylococcus aureus* using disk diffusion antimicrobial method.

**Results:**

The results of GC-MS analysis showed the presence of fatty acids, flavonoids, terpenes, aromatic-aliphatic acids, and their related esters. The total flavonoids in DCM extract of Chenaran, Taleghan, Morad Beyg, and Kalaleh propolis were pinocembrin and pinostrobin chalcone. The common phenolic and terpene compounds detected in all four tested EtOH extracts were *P*-cumaric acid and dimethyl -1,3,5,6-tetramethyl-[1,3-(13C2)] bicycloce [5.5.0] dodeca-1,3,5,6,8,10-hexaene-9,10-dicarboxylate, respectively. The highest inhibitory diameter zone of the Iranian propolis against *C. albicans*, *E. coli*, and *S. aureus* was for DCM extract of Kalaleh propolis (13.33 mm), Morad Beyg propolis (12 mm), and Kalaleh (11.67 mm), respectively.

**Conclusion:**

Iranian propolis showed antimicrobial activities against *C. albicans*, *E. coli*, and *S. aurous*, perhaps due to the presence of flavonoids, phenolic acids, and terpenes as active components that can be used alone or in combination with the selected antibiotics to synergize antibiotic effect, as well as to prevent microbial resistance to available antimicrobial drugs.

## INTRODUCTION

Propolis or bee glue is a resin-like natural material gathered from buds and exudates of plants and mixed with wax by honey bees. Also, bees varnish and sterile the internal walls and the frames of the hive by propolis to prevent the development of microbial diseases in the hive. On the other hand, propolis regulates humidity and temperature in the hive through the year. Since the ancient times, this natural product has been considered by various nations, such as Egyptian, as an antiputrefactive substance to embalme their cadavers[1]. In traditional medicine, propolis has been used as a remedy drug[2]; however, during the last 30 years, it has also been used in hygienic and cosmetic industries, food, and beverages[3,4].

The role of natural products (chemical substances derived from microorganism metabolites or by-products of insects, animals and plants) in medicine and health is significant and hence, it has recently been seen a renewed interest in the use of natural compounds in drug discovery and the development of new therapies against many devastating diseases[5]. Numerous reports have demonstrated the biological activities of various propolis as a global natural product such as antifungal[6-10], antibacterial[11-13], antileishmania[12,14,15], antimalarial[16,17], anticancer [18], anti-inflammatory[19], and antioxidant[20] activities. In this light, several research groups have focused on propolis biological activities and chemical compositions for development of new therapies against various infectious and non-infectious diseases.

It should be noted that the biological activities of propolis depend on its chemical compositions and so far, more than 300 different components of propolis have been reported[4]. It has also been reported that the chemical composition of propolis plays a key role in its biological activities, which may be due to the presence of a wide spectrum of flavonoids, phenolic compounds, aromatic acids, and terpenes that are associated with a variety of health benefits[21,22].

Flavonoids are the main bioactive components of propolis with benzopyranone as the main structure. Various biological activities have been reported for flavonoids[23,24], and their type and amount are mainly associated with the source of plants used by the honey bees. Also, it has been indicated that the highest antimicrobial activity of propolis from Argentina is associated with the high concentration of phenolic and flavonoid compounds[25]. There is a direct effect between chemical composition with vegetation and climate where propolis samples are collected by honey bees. The biological activity of propolis varies and greatly depends on the floral source, as well as the external factors such as season, and environment[26]. In this concern, the analysis of different types of Turkish propolis was evaluated, and the results showed various chemical compositions[27]. Indeed, propolis samples from Marmaris area with Mediterranean climate along with *Populus* spp. and *Salix alba* vegetation and Erzurum with a humid climate were rich in terpenes[27], while those from Bursa with Mediterranean/dry-summer subtropical climate had a high quantity of cinnamyl cinnamate and a low quantity of flavonoids[15]. Previous studies on Iranian propolis from different areas showed a high amount of flavonoid and phenolic compounds that could be responsible for their antimicrobial activities[28-30].

The aforementioned evidence emphasizes the influence of climate and flora diversity on chemical compositions of different types of propolis. To date, the chemical compositions and biological activities of global propolis of many countries have been widely examined[3,31-35] but there is limited investigations related to the quality of Iranian propolis. Therefore, considering the importance of natural products for their remedial and therapeutic value, the purpose of this research was to analyze the chemical compositions of four propolis from different areas of Iran (Chenaran, Taleghan, Morad Beyg, and Kalaleh) with various climates and flora. Moreover, the antimicrobial activity of these four propolis samples was evaluated against *Staphylococcus aureus*, *Escherichia coli*, and *Candida albicans* using the disk diffusion antimicrobial tests.

## MATERIALS AND METHODS

### Chemicals

Hexan, dichloromethane (DCM), ethanol, Bis-(trimethylsilyl)trifluoroacetamide (BSTFA), and trimethylchlorosilane (TMCS) with spectrophotometric grade were purchased from Merck (Darmstadt, Germany). Pyridine and dimethyl sulfoxide (DMSO) were supplied by Sigma-Aldrich (St. Louis, MO, USA). Gentamicin (G10), erythromycin (E15), ketoconazole (KCA 10), and flucytosine (FCN 25) were provided from Mast Group Ltd., UK.

### Propolis collection and origin

**Fig. 1:**
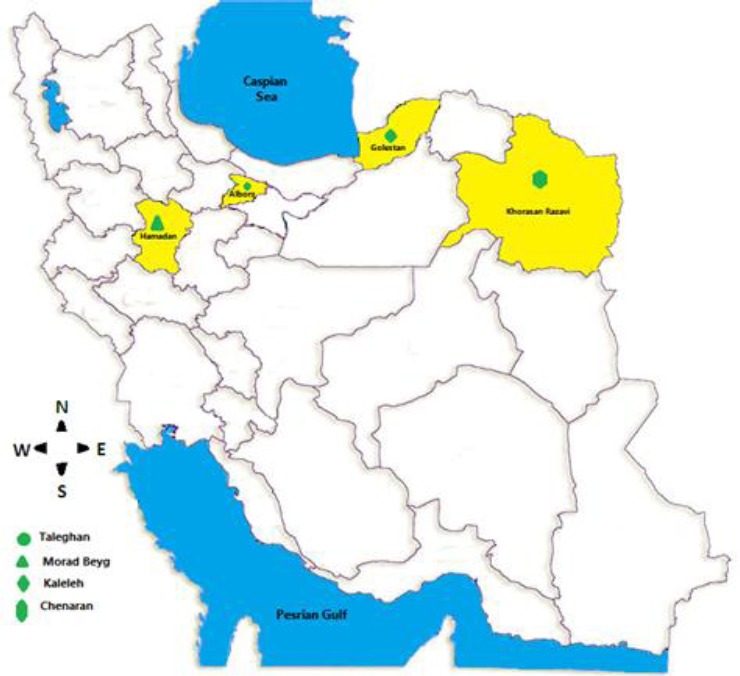
Map of Iran showing the geographic distribution of Iranian propolis studied samples.

In this study, all propolis samples were collected in autumn (August and September 2014) after the honey harvesting season by conventional scraping the frames of *Apis mellifera* bee hives. The study areas with various plant sources were Chenaran in Khorasan-Razavi Province (*Juniperus polycarpus*; 36.64908 N and 59.19118 E), Morad Beyg in Hamedan (*prunus avium* spp. and *populous* spp.; 34.79834N, 48.51497E), Kalaleh in Golestan (poplar plants; 37.37892N, 55.48948E), and Taleghan in Alborz (*Ferula avina*; 36.17307N,50.76946E; Fig. 1). All collected propolis samples were packed into plastic bags and sent to Honey bee Department of Animal Science Research Institute of Iran, where they were cut into the small pieces, protected from light and frozen at 4 °C until the preparation of the extracts.

### Propolis extraction

The propolis samples were powdered by a mortar and pestle, and the powdered samples were mixed with n-hexane with the ratio of 3:100 (w/v; 3 g of crude propolis was mixed with 100 mL of n-hexane) and shaked (120 rpm) at 30 °C for 4 days to remove the bee wax. The mixture was filtered by Whatman 42 filter paper (Sigma-Aldrich Co., St. Louis, MO, USA), and the remaining solid parts of propolis samples on the filter paper were dried at room temperature. After the removal of bee wax, the solid residues were extracted with two different solvents, 70% ethanol (EtOH) and DCM with the ratio of 3:10 (w/v). After hexan extraction, 3 g sample was dissolved in 10 mL of either 70% EtOH or DCM, and the extraction was carried out in the dark on a shaker (120 rpm) at 30 °C for 3 days[36,37]. The 70% EtOH and DCM extract of propolis was filtered by Whatman 42 filter paper under a vacumm. The organic solvent of the filtered extract was removed under reduced pressure at 50 °C for EtOH and 40 °C for DCM extract by a rotary evaporator. The final extracts were stored in a sealed container in a refrigerator at 4 °C and protected from light until gas chromatography-mass spectrometry (GC-MS) analysis and antimicrobial assessment.

### Derivatization procedure

Dried propolis samples (5 g) were mixed in 250-μL pyridine (anhydrous, 99.8%) and 500 μL BSTFA including 1% TMCS (Sigma, Saint Louis, Missouri, USA) in a sealed glass tube at 100 °C for 30 min[38]. Then 1 μL of the prepared samples were injected into GC-MS for analysis.

### Gas chromatography-mass spectrometry analysis

The chemical compositions of the 70% EtOH and DCM extracts of propolis samples (Chenaran, Taleghan, Morad Beyg, and Kalaleh) were characterized by GC-MS analysis. An Agilent 6890N GC equipped with a split/splitless injector, an Agilent 5975C mass selective detector (MSD), and an Auto Sampler CombiPal (CTC analytics, Switzerland) were used. The MS was operated in the electron ionization mode (70 eV). Helium (99.999%) was employed as the carrier gas, and its flow rate was adjusted to 1 mL/min^-1^. The chromatographic separation of chemical compositions was performed on a GC capillary column HP-5MS (30 m×250 μm ID and film thickness of 0.25 μm; J&W Scientific, USA). The initial temperature of the column was set at 40 °C and held for 2 min, then increased by 5 °C min^-1^ to 150 °C and maintained for 3 min, finally increased by 20 °C min^-1^ to 280 °C and held for 10 min. The injector temperature was set at 250 °C in the splitless mode. The split valve was opened after 2 min. The temperature of GC-MS interface, ion source, and quadrupole were set at 280, 230, and 150 °C, respectively. The MS was operated in the scan mode, and the MS scan range was 40-500 atomic mass units. The chromatograms showed a group of peaks that were identified by comparing their retention time and mass spectra. Their mass spectral patterns identified using the Wiley mass spectral library software (version 7n.1) was installed on the GC/MS linked computer.

### Antibacterial activity

A Gram-positive *S. aureus* (ATCC 25923), and a Gram-negative *E. coli* (ATCC 25922), and the yeast *C. albicans* (ATCC 10231) were selected based on their clinical and pharmacological importance. All microorganisms were provided by Department of Microbiology, Pasteur Institute of Iran. Antibacterial and antifungal activities of propolis extracts were investigated by the disk diffusion method[39]. The bacterial and fungal stock cultures were incubated on Mueller-Hinton agar with 5% defibrinated sheep blood (at 37 °C for 24 h) and Sabouraud dextrose agar (Merck, Germany) at 28 °C, respectively, following refrigeration storage at 4 °C. The cell cultures were incubated at 37 °C for 24 h and then used. The cell suspension was adjusted with sterile saline solution to obtain turbidity comparable to that of McFarland no. 0.5 standard (10^10^ cells/mL). The bacterial inoculum was uniformly spread on a sterile nutrient agar plates using sterile cottons swab. Also, all dried extracts of propolis were weighed and dissolved in 70% EtOH and DMSO (10% of the final volume) to obtain 0.1 mg/mL of test samples for antimicrobial analysis. Then, 6-mm filter paper discs (PadtanTeb, Iran) were impregnated with 20 µL of the test samples. The discs were allowed to remain at room temperature until complete diluent evaporation and were kept under refrigeration until ready to be used. Commercial gentamicin (10 µg) and erythromycin (15 µg) for bacteria, as well as KCA 10 µg and FCN 25 µg for the yeast were used as positive controls. However, for negative controls, sterile commercial paper discs (6-mm diameter, PadtanTeb, Iran) were impregnated with 20-μL diluents (70% EtOH and DMSO), which were used to dilute propolis extracts. Discs loaded with propolis and control discs were placed onto the surface of the agar plates. All tests were performed in triplicate. The zones of growth inhibition around the disks were measured after 18-24 h of incubation at 37 °C for bacteria and 24 to 48 h at 28 °C for *Candida albicans*. The sensitivities of the microorganism species to the plant extracts were determined by measuring the sizes of inhibitory zones (including the diameter of disk) on the agar surface around the disks, and the values < 6 mm were considered inactive against microorganisms.

### Statistical analysis

Antimicrobial data *were analyzed* by the *analysis of variance (ANOVA) using SPSS22* statistics. Tukey’s post-hoc test was also used for means separation. *p* < 0.05 was considered statistically significant.

## RESULTS

### Chemical analysis of 70% EtOH and DCM extracts of Iranian propolis

The chemical compositions of 70% EtOH and DCM extracts from four Iranian propolis samples were analyzed by GC-MS. The profiles of the two alcoholic and DCM extracts (appropriate MS data with high probability index >90 were considered) are demonstrated in Table 1 (A and B). Both extracts were composed of aliphatic hydrocarbons, aliphatic acids and its related esters, aromatic acids and its related esters, alkaloids, fatty acids and their related esters, flavonoids, terpenes, sugars, and miscellaneous compounds. Pinocembrin and pinostrobinchalcone were common flavonoids identified in the four DCM proplis extracts.

**Table 1A:** Chemical characterization of 70%EtOH extracts of four Iranian propolis by GC-MS

Components	Composition (%)				Retention Time (min)

	Chenaran	Taleghan	Moradbeig	Klaleh	
**Alkaloids**					
12-Azabicyclo[9.2.2]pentadeca-1(14),11(15)-dien-13-one	-	-	1.62	-	20.71
Oreophilin	-	-	0.35	-	21.24
3’,4’-Dihydro-2’-(morpholin-4-yl)-5’,7’-dinitrospiro[cyclopentane-1,3’-quinazoline]	-	0.14	-	-	26.81
**Aromatic acid and their esters**					
Benzoic acid	0.09	0.11	-	0.01	14.11
Hydroxybenzoic acid	0.27	0.33	-	0.03	16.82
Vanillic acid	-	0.27	-	0.01	17.61
*P*-Coumaric acid	0.19	0.21	0.33	0.02	18.54
Dibutyl phthalate	0.34	0.36	0.34	0.02	18.67
Ferulic acid	-	0.85	0.67	0.03	19.20
Isoferulic acid	0.27	-	0.43	-	19.28
Caffeic acid	-	0.79	1.31	0.08	19.49
2-(2’,4’-Dichloro-phenoxy)phenyl acetic acid	1.96	-	2.33	-	21.82
**Fatty acids and their esters**					
Palmitic acid	6.60	7.22	6.49	0.43	19.01
Margaric acid	0.19	0.19	0.19	0.01	19.46
Oleic acid	1.49	-	0.50	-	19.81
Stearic acid	6.92	7.95	7.01	0.44	19.92
3-Hydroxy stearic acid	0.22	-	-	-	20.68
Eicosanoic acid	0.25	0.25	0.21	0.02	20.83
Behenic acid	-	0.29	0.35	0.04	21.98
Nephrosteranic acid	-	-	0.97	0.08	22.13
2-Methoxycarbonyl-2-(cis-2’pentenyl)-3-methoxy carbonyl cethyl cyclopentane	-	1.13	-	-	23.19
**Flavonoids**					
Osthole	-	0.46	-	0.03	19.61
Pinostrobin chalcone	-	-	0.31	20.90
2’,4’,6’-Trihydroxy chalcone	-	0.99	0.59	21.37
2-(1-(2-Methylcortonoyloxy)-1-methylethyl)-8-oxo-1,2-dihydrofurano[2,3-*H*]2*H*-chromen	-	0.55	-	0.05	22.05
3-Methyl-but-2- enoicacid,2,2- dimethyl-8-oxo-3,4-dihydro-2*H*,8*H*- pyrano[3,2- g]chromen-3-yl ester	-	-	-	0.59	22.33
**Terpenes**					
2*H*-Cyclopentacyclooctene, 4,5,6,7,8,9-hexahydro-1,2,2,3-tetramethyl	0.25	-	-	-	18.57
Germanicol	2.20	3.50	-	-	18.28
Dimethyl -1,3,5,6-tetramethyl-[1,3-(13C2)]bicyclo[5.5.0] dodeca-1,3,5,6,8,10-hexaene-9,10-dicarboxylate	2.51	1.88	2.13	0.14	22.30
Spiro[benzo[a]cyclopenta[3,4]cyclobuta[1,2-c]cycloheptene-8(5*H*),2’-[1,3]dioxane], 6,7,7b,10a-tetrahydro-1	-	1.72	-	-	22.52
14- Methyl-cholest-7-en-3-ol-15-one	0.94	0.76	0.64	-	22.71
(3α,4α)- 4- Methyl- stigmast-22-en-3- ol	1.09	0.70	1.93	-	23.44

**Table 1B:** Chemical characterization DCM extracts of four Iranian propolis by gas GC-MS

Components	Composition (%)				Retention Time (min)
	Chenaran	Taleghan	Moradbeig	Klaleh
**Aromatic acid and their esters**					
Dibutyl phthalate	2.00	1.34	1.83	0.89	18.67
**Fatty acids and their esters**					
Caproic acid	-	-	-	0.16	12.12
Myristic acid	0.7	0.67	0.66	0.50	17.55
Pentadecanoic acid	-	0.31	0.43	0.24	18.08
Methyl palmitate	0.31	-	0.46	-	18.41
Palmitic acid	10.64	8.70	9.45	6.71	19.00
Margaric acid	0.54	0.63	0.70	-	19.09
Methyl stearate	-	-	0.71	-	19.38
Oleic acid	2.86	1.72	2.34	1.30	19.47
Stearic acid	14.59	10.76	10.31	6.69	19.57
2-Methoxycarbonyl-2-(cis-2’pentenyl)-3-methoxycarbonylcethylcyclopentane	-	-	-	0.85	20.29
Sebacic acid, diethyl ester	-	-	-	0.80	23.01
Lignoceric acid	1.16	-	-	-	23.57
**Flavonoids**					
Angecin	-	0.26	-	0.26	17.89
8,8-Dimethyl-2*H*,8*H*-pyrano[2,3-f]chromen-2-one	-	-	-	0.81	19.38
Columbianetin	-	1.00	-	1.70	20.06
Pinostrobin chalcone	1.68	0.53	3.95	1.23	20.88
Pinocembrin	2.71	1.02	4.26	1.38	21.40
2-(1-(2-Methylisocortonoyloxy)-1-methylethyl)-8-oxo-1,2-dihydrofurano[2,3-*H*]2*H*-chromen	-	-	-	1.53	22.06
Tectochrysin	1.51	-	3.23	-	22.10
3-Methyl-but-2-enoic acid, 2,2-dimethyl-8-oxo-3,4-dihydro-2*H*,8*H*-pyrano[3,2-g]chromen-3-yl ester	-	3.88	-	-	22.28
2-Butenoic acid, 2-methyl-, 9,10-dihydro-8,8-dimethyl-2-oxo-2*H*,8*H*-benzo[1,2-b:3,4-b’]dipyran-9-yl ester	-	-	-	0.68	23.35
**Terpens**					
2-Naphthalenemethanol, 1,2,3,4,4a,5,6,7-octahydro-.α.,.α.,4a,8-tetramethyl-, (2R-cis)	0.14	-	-	-	16.96
Agarospirol	-	-	0.24	-	16.97
(1E,3a.α.,7a.β.)-1*H*-Indene, 1-ethylideneoctahydro-7a-methyl	-	-	0.34	-	17.11
1,7,11-Trimethyl-4-(1-methylethyl) cyclotetradecane	-	-	-	0.12	19.20
γ-Sitosterol	-	0.17	-	-	25.20
β-Sitosterol	-	0.18	-	-	26.13
Octahydro cembrene	-	0.12	-	-	26.58

Relevant MS data with high probability index (quality > 90) are shown.

In particular, 70% EtOH extract of Morad Beyg propolis showed the high quantity compounds of different fatty acids and their related esters such as palmitic acid (6.49%) and stearic acid (7.01%). Also, it indicated different cinnamic acid derivatives [ferulic acid (0.67%); isoferulic acid (0.43%); 2-(2’,4’-dichlorophenoxy)phenyl acetic acid (2.33%); caffeic acid (1.31%)], and flavonoid derivatives [pinostrobin chalcone (0.31%); 2’,4’,6’-trihydroxy chalcone (0.59%). The most important terpene derivatives were (3α,4α) - 4-methyl- stigmast-22-en-3-ol (1.93%) and dimethyl -1,3,5,6-tetramethyl-[1,3-(13C2)]bicyclo[5.5.0]dodeca-1,3,5,6,8,10-hexaene-9,10-dicarboxylate (2.13%; Table 1A).

In 70% EtOH, the extract of Taleghan propolis, palmitic acid (7.22%), and stearic acid (7.95%) had the maximum amounts. The extract had different aromatic acid and corresponding esters such as vanillic acid (0.27%), *p*-coumaric acid (0.21%), ferulic acid (0.85%) and flavonoid derivatives [osthole (0.46%); 2’,4’,6’-(trihydroxy) chalcone (0.99%); 2-(1-(2-methylcortonoyloxy)-1-methylethyl)-8-oxo-1,2-dihydrr ofurano[2,3-*H*]2*H*-chromen (0.55%)], as well as various terpene derivatives such as germanicol (3.50%), dimethyl -1,3,5,6-tetramethyl-[1,3-(13C2)] bicycle [5.5.0]dodeca-1,3,5,6,8,10-hexaene-9,10-dicarboxylate (1.88%), and spiro[benzo[a]cyclo penta[3,4]cyclobuta [1,2-c]cycloheptene-8(5H),2’-[1,3] dioxane],6,7,7b,10a-tetrahydro-1 (1.72%; Table 1A).

Similar to Taleghan and Morad Beyg propolis, the 70% EtOH extract of Chenaran propolis had palmitic acid (6.60%) and stearic acid (6.92%) in maximum amounts. Also, different aromatic acids and their esters [*p*-coumaric acid (0.19%); isoferulic acid (0.27%)], as well as terpene derivatives [(3α,4α)-4-methyl-stigmast-22-en-3-ol (1.09%); dimethyl-1,3,5,6-tetramethyl-[1,3-(13C2)] bicyclo[5.5.0]dodeca-1,3,5,6,8,10-hexaene-9, 10-dicarboxylate (2.51%)] were identified (Table 1A). However, galanin (0.99%, quality < 90) was the only identified flavonoid in the ethanolic extract of Chenaran (data not shown).

In comparison to other three EtOH extracts of Iranian propolis, the 70% EtOH extract of Kalaleh propolis had little amounts of palmitic acid (0.43%) and stearic acids (0.44%), and terpene derivative of dimethyl-1,3,5,6-tetramethyl-[1,3-(13C2)]bicycle [5.5.0]dodeca-1,3,5,6,8,10-hexaene-9,10-dicarboxylate (0.14%). The flavonoid compounds [osthole (0.03%); 2-(1-(2-methylcortonoyloxy)-1-methylethyl)-8-oxo-1,2-dihyd-rrofurano[2,3-*H*]2*H*-chromen (0.05); 3-methyl-but-2-enoic acid, 2,2-dimethyl-8-oxo-3,4-dihydro-2H,8H-pyrano[3,2-g]chromen-3-yl ester (0.59%)] were also identified in 70% EtOH extract of Kalaleh propolis (Table 1A).

Regarding the DCM extract of Morad Beyg propolis, the highest quantity compounds were fatty acids, including stearic acid (10.31%) and palmitic acid (9.45%). Other compounds were flavonoids [pinocembrin (4.26%), tectochrysin (3.23%), and pinostrobin chalcone (3.95%)], terpene derivatives [agarospirol (0.24%); (1E,3a.α.,7a.β.)-1*H*-indene,1-ethylideneoctahydro-7a-methyl- (0.34 %)], and dibutyl phthalate (1.83%; Table 1B). Concerning Taleghan propolis, fatty acids [palmitic acid (8.70%) and stearic acid (10.76%)] had the highest amounts, and the other identified compounds were flavonoids [pinocembrin (1.02%); angecin (0.26%); columbianetin (1.00%); 3-methyl-but-2-enoic acid, 2,2-dimethyl-8-oxo-3,4-dihydro-2*H*,8*H*-pyrano[3,2-g]chromen-3-yl ester (3.88%); pinostrobin chalcone (0.53%)], terpene [γ-sitosterol (0.17%); β-sitosterol (0.18%), and octahydro cembrene (0.12%)], and dibutyl phthalate (1.34%; Table 1B).

In case of Chenaran propolis, stearic acid (14.59%) and palmitic acid (10.64%) had the highest quantity. Also, flavanoides [pinocembrin (2.71%); tectochrysin (1.51%), pinostrobinchalcone (1.68%)], terpene derivative [2-Naphthalenemethanol, 1,2,3,4,4a,5,6,7-octahydro-α.,α.,4a,8-tetramethyl-,(2R-cis; 0.14%)], and dibutyl phthalate (2.00%) were identified (Table 1B). In the DCM extract of Kalaleh propolis, fatty acids [palmitic acid (6.71%) and stearic acid (6.69 %)], flavonoids [pinocembrin (1.38%); 2-butenoic acid, 2-methyl-, 9,10-dihydro-8,8-dimethyl-2-oxo-2*H*,8*H*-benzo[1,2-b:3,4-b’]dipyran-9-yl ester (0.68%); columbianetin (1.70%); angecin (0.26%); 8,8-dimethyl-2*H*,8*H*-pyrano[2,3-f]chromen-2-one (0.81%), pinostrobin-chalcone(1.23%); 2-(1-(2-methylisocorto-noyloxy)-1-methylethyl) -8-oxo-1,2-dihydrofurano[2, 3-*H*]2*H*-chromen (1.53%)], terpene derivatives [1,7,11-trimethyl-4-(1-methylethyl) cyclotetradecane (0.12%)], and dibutyl phthalate (0.89%) were identified (Table 1B).

### Comparison of chemical compositions of various propolis in the world

The types of identified flavonoids, phenolic compounds, and terpenes in Iranian propolis and other global countries are shown in Tables 1A and 1B and 2. It is notable that Iranian, Cuban and Brazilian propolis were rich in flavonoids. Also, a high quantity of the phenolic compounds was observed in the propolis obtained from Khojir and Telo (Iran), Minas Gerais (Brazil), and Asyut (Egypt; Table 2). Propolis from Iran, Cuba, and different regions of Brazil had also the high amounts of terpenes (Tables 1 and 2).

**Table 2:** Identified flavonoids, phenolic and terpen compounds in global propolis samples

Country (Reference)	Geographic origin (year)	Flavonoids *	Phenolic compounds*	Terpens*
[28-30]	Lavark	**Pinocembrin**; **kaempferol**; **chrysin**; **galangin**	**Caffeic acid**; **phenethyl caffeate**	
	Khojir (2003)	**Pinostrobin**; **pinocembrin**; pinobanksin-3-acetate; **chrysin**; pinobanksin; **galangin**; **pinocembrin chalcone**; **kaempferol**; pinobanksin-3-propanoate; **quercetin**; quercetin methyl ether	***p*-Coumaric acid**; **dimethyl caffeic acid; isoferulic aci**d; **ferulic acid**; **caffeic acid**; benzyl-*p*-coumarate; 1-phenylethyl trans-caffeate; **cinnamyl caffeate**; methyl-butenyl-ferulate; methyl-butenyl-isoferulate; methyl-butenyl-caffeate; methyl-butenyl-coumarate	δ-9-tetra-Hydrocannabinol acid
	Khojir (2004)	**Pinostrobin chalcone**; **pinocembrin chalcone; pinocembrin**; **chrysin**; **galangin**; **pinobanskin butanoate**; **pinobanskin pentanoate**; pinobanksin acetate; **apigenin**; kaempferol methyl ether; isosakuranetin	***p*-Coumaric acid**; **dimethoxycinnamic acid**; **ferulic acid**; **isoferulic acid**; **caffeic acid**; **penetyl caffeate**; isopentenyl ferulate; dimethylallyl ferulate; **isopentenyl caffeate**; **dimethylallyl caffeate**; **phenethyl caffeate**; **cinnamyl caffeate**	Sesquiterpene; triterpene
	Telo (2003)	**Pinostrobin chalcone**; **pinocembrin chalcone**; **pinocembrin**; **chrysin**; **galangin**; **pinobanskin butanoate**; **pinobanskin pentanoate**; pinobanksin acetate; dihydroxymethoxy flavones; sakuranetin; pinobanskin	***p*-Coumaric acid**; **dimethoxy cinnamic acid**; **ferulic acid**; **isoferulic acid**; **caffeiccid**; pentenyl-*p*-coumarate; beutenyl caffeate; **penetyl caffeate**; **isopentenyl caffeate**; hexyl-*p*-coumarate; **dimethylallyl caffeate**; hexyl caffeate	**Eudesmol**; **α-bisabolol**; diterpenic acid; triterpene
[14,20,49]	Brejo Grande (2010)	6-Acetyl-2,2-dimethyl-3-hydroxy chromen; 2-hydroxy-4-methoxy- chalcone; liquiritigenin; **formononetin**; **medicarpin**; hesperetin 7-rhamno glucoside; biochanin A; retusapurpurin B	-	-
	Brotas, São paulo (2000)	-	Hydrocinnamic acid;***p*-hydroxybenzoic acid**; ***p*-cumaric acid**; **caffeic acid**; *o*-cumaric acid; dihydroxy benzoic acid	**Lupeol acetate**; **lupeol**; lupenone; **lanosterol**; **cycloartenol**; friedour-7-en-3-one; friedour-7-en-3-ol; **α-amyrin**; **β-amyrin**; obtusifoliol; **β-amyrin acetate**
	Marde Espanha (2004)	**Pinostrobin**	**Caffeic acid**, cinnamic acid, ethyl hydro cinnamate, **ferulic acid**, hydrocinnamic acid	**α-Amyrin**; **β-amyrin**; **β-amyrin acetate**; amyrin 3-methoxy; glycyrrhizic acid; patchouli alcohol
[62]	Different provinces (2003-2004)	**Chrysin; pinocembrin; naringenin; sakuranetin; hesperetin; pinobanksin -3-acetate; pinobanksin-3-propionate**	Vanillin; **caffeic acid**; gallaic acid; **ferulic acid**; ferulic acid methyl ester; isoprenyl coumarate; prenyl caffeate; isopentyl caffeate; isoprenyl ferulate; **benzyl caffeate**	-
[51]	Different provinces (2003-2004)	Isoliquiritigenin; liquiritigenin; **formononetin**; vestitol; neovestitol; isosativan; **medicarpin**; homo- pterocarpin; vesticarpan; 3-hydroxy-8, 9-dimethoxy pterocarpan; 3,4-dihydroxy-9-methoxy pterocarpan	-	Nemorosone; 24-methylene-9,19-ciclolanostan-3β-ol; **α-amyrin;** α-amyrone; **β-amyrin; β-amyrinacetat**e; β-amyrone; **cycloartenol**; germanicol; germanicol acetate; **lanosterol**; lanosterol acetate; **lupeol**; **lupeol acetate**
[61]	Baniswief	**Pinostrobin**; **pinocembrin**; **pinobankasin**; **pinobankasin-3-acetate**; **chrysin**; **galangin**	trans-*p*-Coumaric acid; **dimethyl caffeic acid**; **ferulic acid**; **caffeic acid**; **isopentenyl caffeate**; **dimethyl allyl caffeate**; **benzyl caffeate**; dodecyl caffeate; tetradecylcaffeate; hexa decylcaffeate; tetradecenyl caffeate	Cycloartinol; **lanosterol**; **β-amyrin**; **triterpene of β-amyrin type**
	Fayoum	**Pinostrobin chalcone**; **pinocembrin chalcone**; **galangin**; **chrysin**; Sakauranetin chalcone; **pinobankasin; pinobankasin-3-acetate**	**diMethylcaffeic acid**; **caffeic acid**; **isopentenyl caffeate**; 2-methyl-2-butenyl caffeate	-
	Asyut	**Pinostrobin chalcone**; **pinocembrin**; **pinobankasin**; **chrysin**	4-Methoxy-cinnamic acid; **dimethyl caffeic acid**; **isoferulic acid**; **caffeic acid**;different derivatives of methyl-butenyl-coumarate;**isopentenylcaffeate**; 2-methyl-2-butenyl caffeate;3-methyl-2-butenyl caffeate	-
	Shouhag	Hexamethoxy flavone	***p*-Hydroxy benzoic acid**; **dimethyl caffeic acid**; **caffeic acid**	**Dehydroabietic acid**;**β-amyrin**; **triterpene of β-amyrin type**
[13,15,27,54]	Erzurum (Anatolia)	**Naringenin**; **chrysin;** acacetin	**Ferulic acid**; **dimethyl caffeic acid**; **isoferulic acid**; 4-vinylphenol; 2-Methoxy-4-vinyphenol	**Chrysophanol**; α-cadinol **β-eudesmol**; **α-bisabolol**; α-eudesmol; 2-naphtalenemethanol
	Bursa and Hatay (2007)	**Tectochrysin**; **pinocembrin**; **chrysin**; 4*H*-1-benzopyran-4-one, 3,5,7-trihydroxy-2-phenyl	Methylhomovanillate; **isoferulic acid**	Totarolone; hinokione; bicyclo(4.4.0) dec-1-ene; δ-cadinene
	Different provinces (2002-2003)	Isalpinin; **pinocembrin**; **pinostropin**; **naringenin**; 4´,5-dihydroxy-7-methoxy flavanone; **chrysin**; 3,4´,7-trimethoxy flavanone; **pinobanksin**; **quercetin**;**** **galangine**; **apigenin**	**Ferulic acid**; **caffeic acid**; **isoferulic acid**	5-Azulenementhanol; **α-bisabolol**
	Kazan and marmaris (1996)	-	**Ferulic acid**; **caffeic acid**; caffeic acid isomers	1-Naphthalene methanol, decahydro-1,10-dimethyl-6-methenyl-5-(5-hydroxy-3-Pentene); thunbergol; **isopimaric acid**; 3-α,5-β-pregnan-20-one; androstan-1,17-dimethyl-17-hydroxy-3-one; α-terpineol; 4-β*H*,5α-eremophi1D1(10)-ene; **dehydroabietic acid**; abietic acid; farnesol
[14]	Burgas (2003)	**Pinobanksin 3-butanoate**; pinobanksin 3-etanoate; **pinostrobin chalcone**; **chrysin**; **pinobanksin 3-pentanoate**; **pinocembrin**	**Benzyl caffeate**; **caffeic acid**; **dimethyl caffeic acid**; **ferulic acid**; **isoferulic acid**;***p*-coumaric acid**; pentenyl caffeate; pentenyl ferrulate; **phenethylcaffeate**	Squalene

* The bold compositions are common between different propolis samples collected from global regions. In comparasion with Table 1 (A and B), Iranian propolis from Chenaran, Taleghan, Morad Beyg, and Kalaleh (the present study) are rich in flavonoids, phenolic, and terpenes compounds similar to Cuban, Brazilian, and Egyptian propolis.

### Antimicrobial activity

As compared with standard drugs, the growth inhibition zone for 70% EtOH extract of Iranian propolis ranged between 8.33 and 10 mm for gram-negative *E. coli*; however, this range was between 10.33 and 12 mm for its DCM extract (Table 3). The observed inhibition zone of microbial growth diameter of all propolis samples for gram-positive *S. aureus* presented an inhibition zone of 8.67- 10 mm for 70% EtOH and 10.33-11.67 mm for DCM extracts (Table 3). For antifungal activity against *C. albicans*, the zone of inhibition was 9-11 mm for 70% EtOH and 10-13.33 mm for DCM extracts (Table 3). The DCM extracts of Morad Beyg (12 mm) and Kalaleh (11.67 mm) showed the highest antibacterial activity against *E. coli* and *S. aurous*, respectively. The evaluation of antifungal activity of these extracts also presented the best result for Kalaleh DCM extract against *C. albicans* with an inhibition zone of 13.33 mm. The negative controls (70% EtOH and DMSO) did not show any antibacterial and antifungal activity. Reference drugs indicated higher antimicrobial activity in comparison to all extracts; erythromycin and gentamicin with 16.33 and 19.33 mm inhibition zone against *E.coli* and 17 and 20.67 mm against *S. aurous*, respectively. Also, the inhibition zones for KCA and FCN against *C. albicans* were 13.67 and 19.67 mm.

**Table 3:** Antibacterial and antifungal activities of %70 EtOH and DCM extracts of propolis from different areas of Iran

Samples	Inhabitation zone of microbial growth diameters (mm)					

	*E. coli* ATTC25922		*S. aurous* ATCC6538		*C. albicans* ATCC10231	
		
	70% EtOH	DCM	70% EtOH	DCM	70% EtOH	DCM
Chenaran (Mashhad)	8.33^e^	11^cd^	8.67^g^	11.00^cde^	9.00^f^	10.33^de^
MoradBeyg (Hamedan)	10.00^d^	12^c^	10.00^def^	11.33^cd^	11.00^de^	12.00^cd^
Kalaleh (Goleatan)	9.00^de^	11.33^c^	10.00^def^	11.67^c^	10.67^de^	13.33^bc^
Alborz (Taleghan)	9.00^de^	10.33^d^	9.33^efg^	10.33^cdef^	10.33^ef^	11.67^d^
Erythromycin 15	16.33^b^		17.00^b^		13.67^b^	
Gentamicin 10	19.33^a^		20.67^a^		19.67^a^	
Ketoconazole (KCA 10)						
Flucytosine (FCN25)						
DMSO	-		-		-	
70% EtOH	-		-		-	

Values are mean of three different tests. Means followed by the same letters are not significantly different (Tukey’s test; *p* < 0.05). - shows no zone of inhibition

## DISCUSSION

In recent years, due to antimicrobials failure, there has been a growing interest in research to find effective antimicrobial agents from various sources. In this light, many researchers have focused on natural products as a source of new bioactive molecules. Propolis is one of the promising natural products with antimicrobial activities. These bioactivities surely depend on its chemical compositions. Therefore, in the current study, chemical compositions of four Iranian propolis samples were analyzed using GC-MS methods. Furthermore, more than 250 and 150 individual compounds were identified in 70% EtOH and DCM extractions, respectively. GC-MS analysis showed that the identified compounds belonged to different groups of chemicals such as fatty acids, phenolic acids, and their related esters, flavonoids, alkaloids, aliphatic hydrocarbons, aliphatic acids and their related esters, and terpenes.

Indeed, the common compounds in all 70% EtOH extracts of Iranian propolis were fatty acids (palmitic acid, stearic acid, eicosanoic acid, and margaric acid), aliphatic esters (lactic acid, glycolic acid, succinic acid, malic acid [data not shown]), aromatic acid (*p*-coumaric acid), aromatic ester (dibutyl phthalate), and dimethyl-1,3,5,6-tetramethyl-[1,3-(13C2)] bicycloe [5.5.0]dodeca-1,3,5,6,8,10-hexaene-9,10-dicarboxylate, while some of the compounds were only identified in a limited number of tested propolis. However, the common compounds in all tested DCM extracts of Iranian propolis were fatty acids (myristic acid, palmitic acid, stearic acid, and oleic acid), as well as flavonoids (pinocembrin and pinostrobinchalcon), and the remaining compounds were identified in one, two, or three out of four tested samples. Moreover, pinostrobin chalcone was identified in both 70% EtOH and DCM extracts of Morad Beyg propolis (0.31% and 3.95% respectively). Since all four tested propolis samples collected from areas with various climate and flora, the present results confirm the direct effect of vegetation and climate on chemical composition of propolis.

In this work, propolis of Morad Beyg (11.44%) and Kalaleh (7.59%) had the highest content of flavonoids in their DCM extracts; however, in the 70% EtOH extracts, phenolic compounds were high. Also, terpenes had the highest quantity in 70% EtOH extract of Taleghan (8.56%), Chenaran (6.99%), and Morad Beyg (5.67%) propolis samples but Kalaleh propolis showed low amount (0.14%) of this compound. It is worth mentioning that solvent and extraction method could play a key role in the isolation of bioactive compounds. In this concern, the comparison between two utilized extractions in this study also indicated that the total amount of flavonoids in DCM extracts of propolis collected from four regions was higher than ethanolic extracts. However, the 70% EtOH was more efficient solvent for the isolation of phenolic compounds compared to DCM. Terpenes were identified in both 70% EtOH and DCM due to their hydrophobic characteristics. Therefore, it is expected that based on the higher prevalence of flavonoides in the DCM extracts of Morad Beyg and Kalaleh popolis, higher biological activities should be obsereved in comparison with the other two propolis. In fact, the antimicrobial activities of DCM extract of Morad Beyg propolis against *E. coli* and the DCM extract of Kalaleh propolis against *S. aurous* and *C. albican* confirm this assumption.

As reported previously, the antimicrobial activity of propolis is due to flavonoids and aromatic acids and their esters such as galangin, pinocembrin, pinostrobin as well as ferulic and caffeic acid[40-42]. This view could explain the antimicrobial activities of Iranian propolis that may be because of the presence of the most effective flavonoid agents, including pinocembrin and pinostrobinchalcone (DCM: all four propolis) along with aromatic acid, ferulic (EtOH: Talegan and Morad Byge), and caffeic (EtOH: Morad Beyg) acids, which contribute to the bactericidal action of propolis[37]. Based on this result, it seems that the antimicrobial activities of Iranian propolis could be associated with a combination of or/and synergism between flavonoids, aromatic acids, and terpenes[43-45]. This antimicrobial (antibacterial and antifungal) activity of propolis has also been reported for propolis from Kenya[46], Egypt, China, Bulgaria, Spain, Australia, Greece, Italy[47], Brazil (Goiás, Paraná and São Paulo States)[48-50], Cuba[51], Portugal (Bragança county)[17], Bosnia and Herzegovina[52], Serbia[53], and Turkey[54-56]. Interestingly, antifungal aspect of ethanolic propolis extracts of Iranian propolis from Azerbijan and Kerman provinces was reported against *C. albicans*[10]. In addition to antibacterial and antifungal activities of global propolis, antiprotozoal properties were also reported for propolis from Cuba[51], Bulgaria[14], Turkey (Bursa)[15], Portugal (Bragança county)[17], Brazil[16], and Java[57].

In the current study, the inhibitory diameter zone of the Iranian propolis against *E. coli* ranged from 8.33 to 12 mm, and the highest was for DCM extract of Morad Beyg propolis (12 mm). The inhibitory diameter zones against *S. aureus* were from 8.67 to 11.67 mm, and DCM propolis extract of Kalaleh showed the highest antimicrobial activity (11.67 mm). However, the antibacterial activity of ethanolic extracts of Kalaleh and Morad Beyg propolis against *E. coli* (current study) was higher than that previously reported for Iranian propolis[29,30]. In 70% EtOH extract of Iranian propolis samples, no significant differences were observed between all propolis samples against *E. coli* (*P*≥0.05). In case of DCM extract of Iranian propolis, there were no significant differences among Chenarn, Morad Beyg, and Taleghan propolis samples against *S. aurous* (*P*≥0.05)*;* however, Kalaleh propolis indicated significant activity (*P*≤0.05). The inhibitory diameter zone against *C. albicans* was in range of 9-13.33 mm, and the highest range was for DCM extract of Kalaleh propolis (13.33 mm), which was almost similar to KCA (13.67 mm). It should be noted that both 70% EtOH and DCM extract of Kalaleh propolis showed highest activities against *S. aureus*, *E. coli*, and *C. albicans*; however, the Brazilian propolis has been demonstrated to have antifungal activity against *C. albicans*, but not *E. coli* [50]. The same report from 30% EtOH extract of Iraqi propolis indicated antibacterial activities against *S. aureus* and *E. coli* (MIC; 640 and 1280 µg/mL) but there was no report of antifungal activity against *C. albicans*[58]. In general, this discrepancy in antimicrobial activities of global propolis supports and confirms the difference in flavonoids, phenolic, and terpeneoids compounds as all these compounds were responsible for biological activities, which is associated with variation in flora, climate, and season of the studied areas[59,60].

It should be also noted that the type and the amount of propolis components are dependent on the plant source where honey bees use at the site of collection of propolis, and so far the most reported plant species used by hony bees are *poplars* (*Populus* spp.), *Pinus brutia*, *Tipuana tipu, Baccharis dracunculifolia*, *Salix alba*, and Cypress family. Probably, difference between chemical compositions of four Iranian propolis samples depends on flora in Chenaran (*Juniperus polycarpus*), Morad Beyg (*Prunus avium* spp. and *Populous* spp.), Kalaleh (poplar plants), and Taleghan (*Ferula avina*). In an earlier work in Brazil, pentacyclic triterpenes, flavonoides, diterpenes, and cinnamic acid derivatives were identified in *Baccharis dracunculifolia* extracts, a plant source for the Brazilian green propolis[16]. This type of Brazilian propolis showed antileishmanial and antiplasmodial activities. Also, in Cuban propolis, the major compositions were flavonoid (vestitol, neovestitol, and isosativan) and terpene (cycloartenol, amyrin, and lupeol) compounds, which both indicated antimicrobial activities[51]. In Egypt, in an area with the plant source of *Populus* spp., collected propolis indicated the antimicrobial activities, which could be associated with aliphatic, aromatic acids and their related esters, flavonoids, and terpenes compounds[60]. In Turkish propolis (with *Pinusbrutia* flora), the most chemical compositions were phenolics, terpenes, aliphatic and aromatic acids, and their related esters[27]. Also, in Bulgarian propolis (with Populus *nigra* flora), the highest amount of pinobanksin 3-butanoate (9.85%), pinobanksin 3-etanoate (11.23%), pinocembrin (9.44%), and squalene (4.41%) were detected in the ethanolic extract[14]. Moreover, in a recent work by Al Naggar and co workers[61], 70% ethanolic extracts of Canadian propolis from various regions showed different chemical compositions containing coumaric acid, ferulic acid, caffeic acid, benzyl caffeate, pinocembrin, sakuranetin, and pinobanksin-3-acetate. All aforementioned reports as well as the report from the current study support the direct effect of vegetation and climate on propolis compositions and biological activities.

In summary, propolis research has become the subject of intense discovery of new and novel bioactive compounds, and in this concern, 70% EtOH and DCM extracts of four types of Iranian propolis were characterized for the first time by GC-MS analysis. Iranian propolis showed antimicrobial activities against *C. albicans*, *E. coli*, and *S. aurous*, perhaps due to the presence of flavonoids, phenolic acids, and terpenes as active components. However, further research is highly required to isolate and identify active compound(s) from propolis and standardize this honey bee product. These active compounds of propolis can be used alone or in combination with the selected antibiotics (ampicillin, ceftriaxone, doxycycline, amikacin, nalidixic acid, and trimethoprim/sulfamethoxazole) to synergize antibiotic effect as well as to prevent microbial resistance to available antimicrobial drugs.
